# The Association Between Personality and Homebound Status in Older Adults: Results From the NHATS

**DOI:** 10.1093/geroni/igaa057.751

**Published:** 2020-12-16

**Authors:** Xiaocao Sun, Minhui Liu, Christina E Miyawaki, Yuxiao Li, Tianxue Hou, Siyuan Tang, Sarah Szanton

**Affiliations:** 1 Central South University, Changsha City, Hunan, China; 2 Central South University, Baltimore, Maryland, United States; 3 University of Houston, Seattle, Washington, United States; 4 Central South University, Changsha, Hunan, China; 5 Johns Hopkins University, Baltimore, Maryland, United States

## Abstract

Personality is associated with predictors of homebound status like frailty, incident falls, and depression. It has been rarely investigated whether personality predicts homebound status among older adults. Using the combining cross-sectional data of the Year 2013 and Year 2014 data from the National Health and Aging Trends Study (NHATS), this study examined the association between personality traits and homebound status in a sample of community-dwelling older adults aged 65 years and older (N=2,788). Homebound status (non-homebound, semi-homebound, and homebound) was determined by the frequency, difficulty, and help of outdoor mobility. Personality traits, including conscientiousness, agreeableness, openness, extraversion, and neuroticism were assessed using the 10-item Midlife Development Inventory on a rating scale from 1 (not at all) to 4 (a lot). Each personality trait was included as a predictor in an ordinal logistic regression model to examine its association with homebound status after adjusting demographic and health-related covariates. The sample was on average 79±7.53 years old, non-Hispanic White (72.0%), female (58.6%), living alone (35.4%) or with spouse/partner only (37.4%). Seventy-four percent, 18%, and 8% of participants were non-homebound, semi-homebound, and homebound, respectively. Homebound participants tended to be less-educated older females. The average scores of conscientiousness, agreeableness, openness, extraversion, and neuroticism were 3.19±0.75, 3.57±0.56, 2.81±0.83, 3.13±0.75, and 2.22±0.86, respectively. Among these five personality traits, high conscientiousness (OR=1.34, p<0.001) and extraversion (OR=1.16, p=.03) were associated with a reduced likelihood of being homebound. These findings provided a basis for potential personality assessment to identify and protect individuals with high homebound risk.

